# FEASIBILITY AND EFFICACY OF THE LEAD DEMENTIA ADVANCE CARE PLANNING INTERVENTION

**DOI:** 10.1093/geroni/igad104.0426

**Published:** 2023-12-21

**Authors:** Kara Dassel, Eli Iacob, Jordana Clayton, Nancy Aruscavage, Katherine Supiano, Rebecca Utz

**Affiliations:** University of Utah College of Nursing, Salt Lake Cty, Utah, United States; University of Utah, Salt Lake City, Utah, United States; University of Utah College of Nursing, Salt Lake City, Utah, United States; University of Utah College of Nursing, Salt Lake City, Utah, United States; University of Utah, Salt Lake City, Utah, United States; University of Utah, Salt Lake City, Utah, United States

## Abstract

Using our dementia-focused advance care planning tool (ACP), the LEAD Guide (Life-Planning in Early Alzheimer’s and Other Dementias), we implemented a 9-month NIH Stage-1 behavioral intervention to promote ACP concordance in community-based dementia dyads. Baseline concordance was assessed using individual responses on the LEAD Guide (patient for themselves, care partner as if they were the patient). Dyads compared responses, discussed discrepancies, and the patient revised their responses. Concordance for values (quality of life, burden) and preferences (care location and life-sustaining treatments) across the disease trajectory was calculated using cross-tabs. At baseline, dyadic discordance was less than 10% on quality-of-life measures except for whether the patient would prefer a longer but less satisfying life (23.8%); however, post-conversation, 5.9% of patients changed their responses. About a fifth of the dyads showed discordance regarding financial, emotional, and physical burden (19.0%, 19.1%, and 16.7%, respectively); however, post-conversation, a few patients changed their responses (14.6%, 8.8%, and 5.9%). At baseline, discordance on questions for life-sustaining treatments across the disease trajectory varied; ventilator (now 14.3%, future 26.2%), brain dead (16.7% now and future), feeding tube (30.1% now, 28.6% future), and severe pain (23.8% now, 21.4% future). Following conversations, a few patients’ changed their responses on these items (8.8%, 12.1%, 8.8%, and 12.1%). Higher discordance on value questions on burden compared to the quality of life may result from care partners’ bias. Fluctuations in concordance regarding life-sustaining treatments in the present versus future states of advanced dementia demonstrate the need to engage in more in-depth care conversations.

